# Breast cancer cells stimulate osteoprotegerin (OPG) production by endothelial cells through direct cell contact

**DOI:** 10.1186/1476-4598-8-49

**Published:** 2009-07-15

**Authors:** Penny E Reid, Nicola J Brown, Ingunn Holen

**Affiliations:** 1 Academic Units of Clinical Oncology and Surgical Oncology, School of Medicine and Biomedical Sciences, University of Sheffield, Beech Hill Road, Sheffield, UK

## Abstract

**Background:**

Angiogenesis, the sprouting of capillaries from existing blood vessels, is central to tumour growth and progression, however the molecular regulation of this process remains to be fully elucidated. The secreted glycoprotein osteoprotegerin (OPG) is one potential pro-angiogenic factor, and clinical studies have demonstrated endothelial cells within a number of tumour types to express high levels of OPG compared to those in normal tissue. Additionally, OPG can increase endothelial cell survival, proliferation and migration, as well as induce endothelial cell tube formation *in vitro*. This study aims to elucidate the processes involved in the pro-angiogenic effects of OPG *in vitro*, and also how OPG levels may be regulated within the tumour microenvironment.

**Results:**

It has previously been demonstrated that OPG can induce tube formation on growth factor reduced matrigel. In this study, we demonstrate that OPG enhances the pro-angiogenic effects of VEGF and that OPG does not stimulate endothelial cell tube formation through activation of the VEGFR2 receptor. We also show that cell contact between HuDMECs and the T47D breast cancer cell line increases endothelial cell OPG mRNA and protein secretion levels in *in vitro *co-cultures. These increases in endothelial cell OPG secretion were dependent on α_ν_β_3 _ligation and NFκB activation. In contrast, the pro-angiogenic factors VEGF, bFGF and TGFβ had no effect on HuDMEC OPG levels.

**Conclusion:**

These findings suggest that the VEGF signalling pathway is not involved in mediating the pro-angiogenic effects of OPG on endothelial cells *in vitro*. Additionally, we show that breast cancer cells cause increased levels of OPG expression by endothelial cells, and that direct contact between endothelial cells and tumour cells is required in order to increase endothelial OPG expression and secretion. Stimulation of OPG secretion was shown to involve α_ν_β_3 _ligation and NFκB activation.

## Background

Angiogenesis, the sprouting of capillaries from existing blood vessels, is central to tumour growth and progression and the balance between pro-angiogenic and anti-angiogenic factors is thought to regulate this process [1]. Factors such as vascular endothelial growth factor (VEGF), fibroblast growth factor (FGF) and the angiopoietins are well-established promoters of angiogenesis. However, the molecular regulation of tumour angiogenesis remains to be fully elucidated [2].

One potential pro-angiogenic factor is osteoprotegerin (OPG) [3,4]. OPG is a secreted glycoprotein belonging to the tumour necrosis factor receptor (TNFR) superfamily, initially identified for its role in regulating bone turnover through the binding and neutralisation of receptor activator of NFκB ligand (RANKL). Subsequently OPG has been found to have additional roles within the immune and vascular systems, as well as promoting tumourigenesis [5]. Observations that OPG deficient mice exhibit vascular calcification provided initial evidence that OPG could have a role in the vascular system and further *in vivo *studies have demonstrated the involvement of OPG in vascular complications, including atherosclerotic plaque calcification [6-8]. These findings have been supported clinically, with both OPG polymorphisms and increased serum levels being associated with an increased risk of coronary artery disease [9-11]. Additionally, OPG has been associated with other vascular complications, including ischaemic stroke and pulmonary arterial hypertension [12,13]. With reference to *in vitro *studies OPG has been found to increase endothelial cell survival, proliferation and migration, as well as induce endothelial cell tube formation in an *in vitro *matrigel model of angiogenesis [3,14]. Recently, α_ν _integrin has been found to be involved in OPG-induced endothelial cell migration and proliferation, however mechanisms for other potential pro-angiogenic effects such as OPG-stimulated tube formation remain to be established [14].

Clinical studies have shown endothelial cells within a number of tumour types to express high levels of OPG compared with those in normal tissues, and in breast cancer this expression was found to correlate with tumour grade [3]. *In vitro*, endothelial cells have been found to secrete OPG capable of inhibiting tumour necrosis factor (TNF)-related apoptosis inducing ligand (TRAIL)-induced apoptosis of breast cancer cells, indicating endothelial-derived OPG to be functionally active [3]. Other studies have demonstrated the ability of OPG to inhibit TRAIL-induced apoptosis of a variety of cancer cell lines [15-17]. Therefore, it is possible that OPG can promote tumourigenesis both directly, via pro-survival actions on tumour cells and also indirectly, through the stimulation of angiogenesis. Previous studies have found endothelial cell OPG levels to be up-regulated in response to pro-inflammatory factors including IL-1α and TNFα [18]. However, processes involved in regulating endothelial OPG levels in the tumour microenvironment are currently unknown.

Therefore, this study aims to address two key points. Firstly, to elucidate the mechanisms behind the pro-angiogenic effects of OPG and secondly, to establish whether this is relevant in the tumour microenvironment.

## Results

### Effect of VEGF in combination with OPG on endothelial cell tube formation

**Figure 1:**
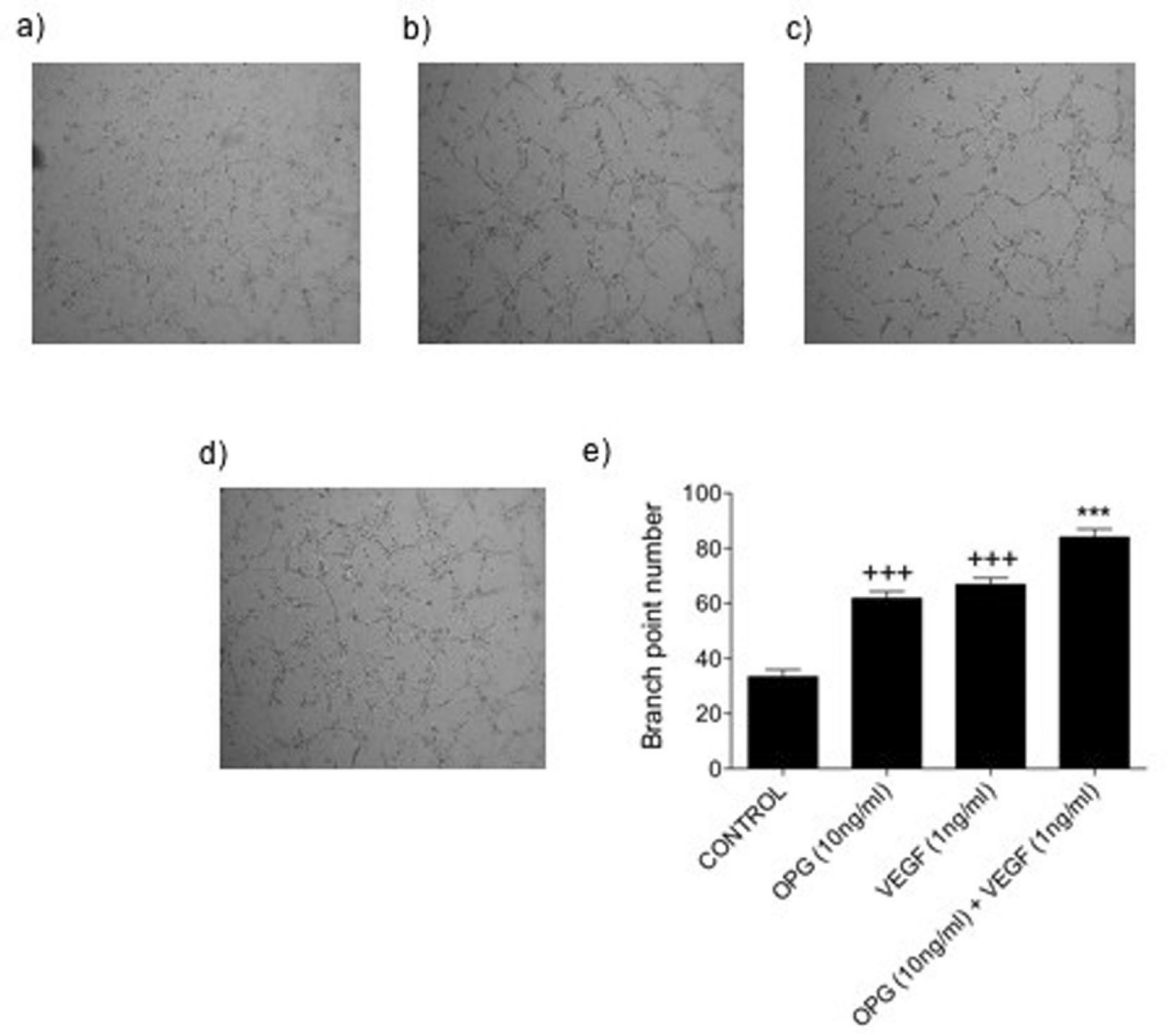
**Effect of VEGF in combination with OPG on endothelial cell tube formation**. HuDMECs were cultured on growth factor reduced matrigel for 8 hours and branch point number counted as described in materials and methods. (a) Untreated control, (b) OPG 10 ng/ml, (c) VEGF 1 ng/ml, (d) OPG 10 ng/ml and VEGF 1 ng/ml. (e) Quantification of tube formation through measurement of branch point number. Data represented as mean ± S.E.M. from three independent experiments performed in triplicate. ***, *p *< 0.001 compared to VEGF (1 ng/ml), OPG (10 ng/ml) or untreated control; +++, *p *< 0.001 compared to untreated control only.

It has previously been shown that OPG can induce endothelial cell tube formation on growth factor reduced matrigel [3]. However, in the tumour microenvironment it is possible that OPG also enhances vessel formation induced by other pro-angiogenic factors such as VEGF. Therefore, to further elucidate how OPG may affect tube formation, endothelial cells were treated with a combination of OPG and VEGF to establish whether the two have a synergistic effect on tube formation *in vitro*. As demonstrated in figure 1, OPG or VEGF when administered alone significantly increased tube formation, almost doubling the number of branch points (*p *< 0.001). When OPG and VEGF were added together, tube formation was significantly increased by 20% compared to VEGF alone (*p *< 0.001) and 26% compared to OPG alone (*p *< 0.001). This suggests that within the tumour microenvironment OPG is able to act in concert with other pro-angiogenic factors such as VEGF to further enhance angiogenesis and additionally, that OPG and VEGF act via different pathways to induce endothelial cell tube formation.

**Figure 2:**
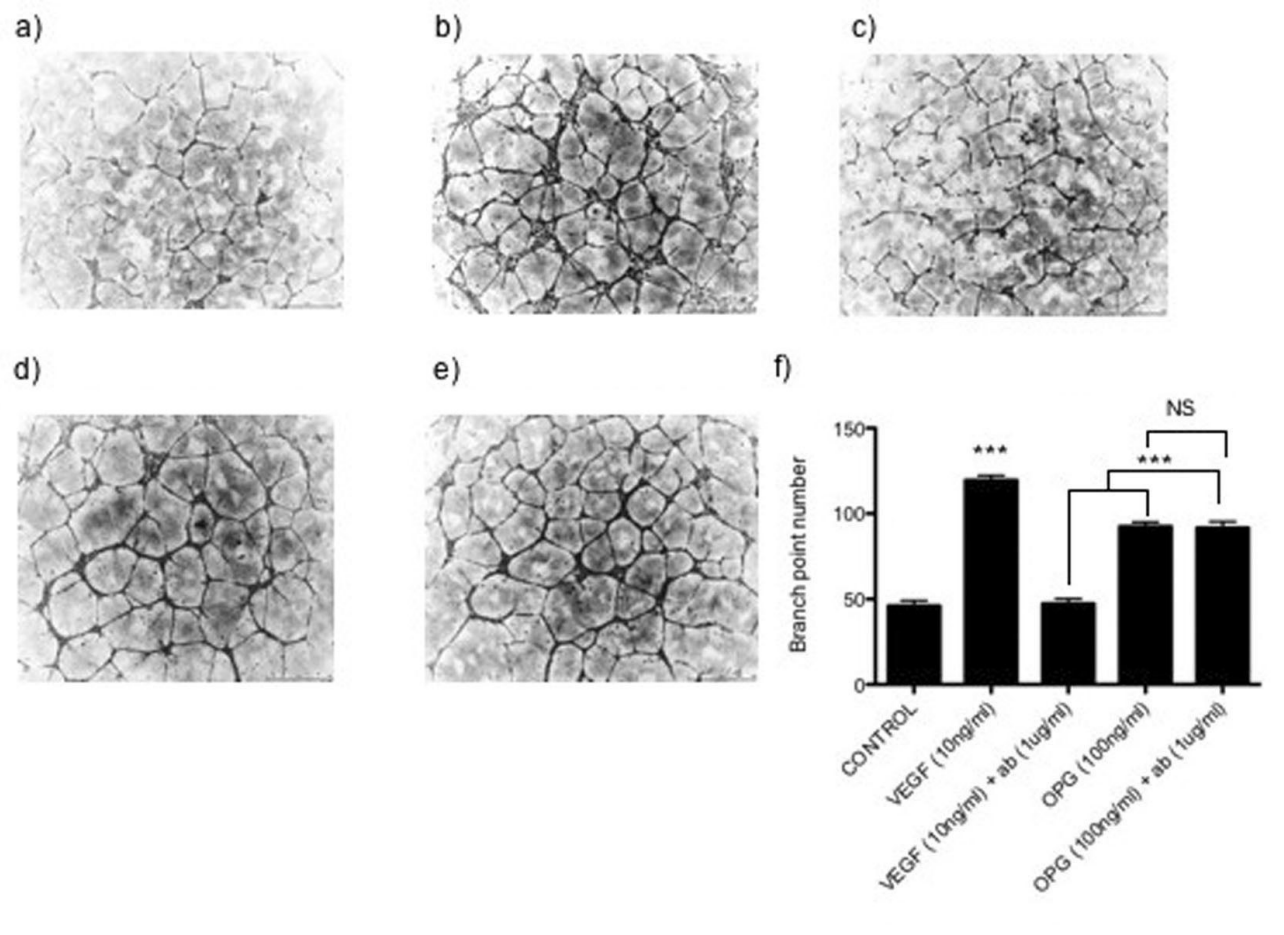
**Involvement of the VEGF receptor VEGFR2 in OPG mediated endothelial tube formation**. HuDMECs were cultured on growth factor reduced matrigel for 8 hours and branch point number counted as described in materials and methods. (a) Untreated control, (b) VEGF 10 ng/ml, (c) VEGF 10 ng/ml and VEGFR2 neutralising antibody (1 μg/ml), (d) OPG 100 ng/ml, (e) OPG 100 ng/ml and VEGFR2 neutralising antibody (1 μg/ml). (f) Quantification of tube formation through measurement of branch point number. Data represented as mean ± S.E.M. from three independent experiments performed in triplicate. ***, *p *< 0.001 compared to VEGF in combination with VEGFR2 antibody; NS, no significant difference.

To confirm this, the levels of HuDMEC tube formation induced by either OPG or VEGF were determined in the presence or absence of a VEGFR2 neutralising antibody. As demonstrated in figure 2, both VEGF and OPG stimulated HuDMEC tube formation on growth factor reduced matrigel compared to control. VEGF-induced tube formation was inhibited in the presence of the anti-VEGFR2 antibody as observed by a 60% decrease in branchpoint number (*p *< 0.001). In contrast, administration of the anti-VEGFR2 antibody had no effect on OPG-induced tube formation, suggesting that OPG does not induce tubule formation through interacting with the VEGFR2 receptor (Figure 2).

### Effect of pro-angiogenic factors on endothelial cell OPG production

**Figure 3:**
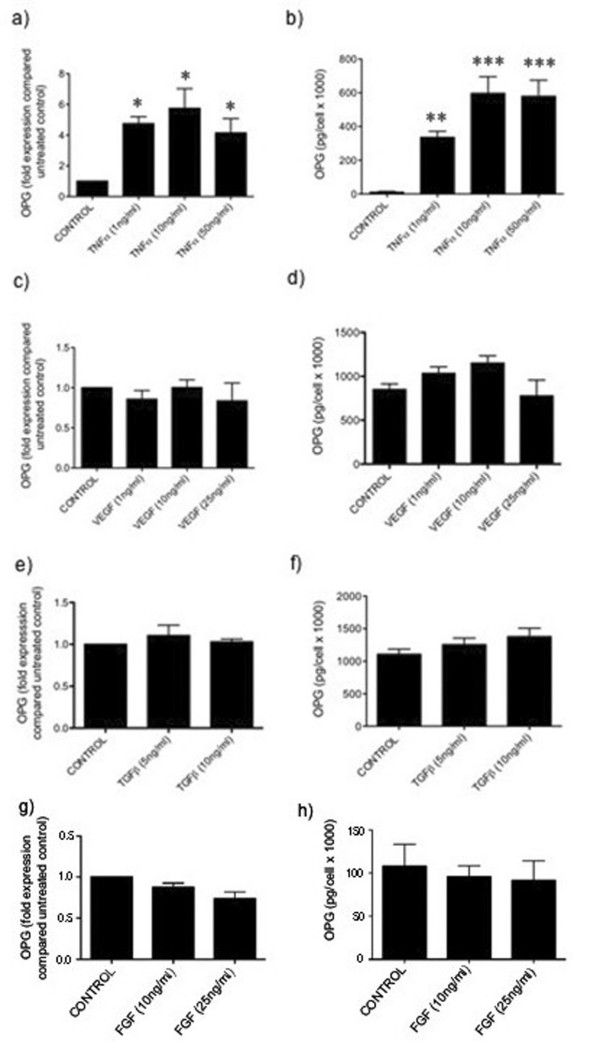
**Effect of pro-angiogenic factors on endothelial cell OPG production**. HuDMECs were treated with increasing concentrations of TNFα (a) and (b), VEGF (c) and (d), TGFβ (e) and (f), or FGF (g) and (h) for 24 hours. Conditioned medium was collected and OPG secretion measured using ELISA as described in materials and methods. RNA was extracted and gene expression quantified using real-time PCR. For real-time quantitative PCR, values were normalised to GAPDH and are given as fold expression compared to untreated HuDMECs. Data are represented as mean ± S.E.M. from three independent experiments performed in triplicate. ***, *p *< 0.001 compared to untreated control; **, *p *< 0.01 compared to untreated control; *, *p *< 0.05 compared to control.

One potential mechanism by which endothelial cell OPG production may be elevated within the tumour microenvironment is through endothelial cell stimulation by pro-angiogenic factors. To assess the ability of pro-angiogenic factors to affect the levels of endothelial OPG production, HuDMECs were treated for 24 hours with VEGF (1–25 ng/ml), TGFβ (5–10 ng/ml) or FGF (10–25 ng/ml), as well as the pro-inflammatory cytokine TNFα (1–50 ng/ml), which has previously been shown to stimulate endothelial OPG production [18,19]. Levels of gene expression were then determined using real-time PCR and OPG secretion assessed using ELISA. As demonstrated in figure 3(a), TNFα significantly increased HuDMEC OPG gene expression and at 10 ng/ml, expression levels were 6-fold greater than untreated HuDMECs (*p *< 0.05). Similarly, OPG secretion levels were significantly increased (*p *< 0.001 at 10 ng/ml and 50 ng/ml) (Figure 3(b)), confirming previous studies in endothelial cells [18-20]. In contrast, no significant change in OPG expression or secretion was detected after HuDMEC were treated with VEGF, TGFβ or FGF (Figure 3(c), (d), (e), (f), (g) and 3(h)). This suggests that some of the key pro-angiogenic factors do not have a central role in enhancing endothelial cell OPG levels.

### Effects of tumour cells on endothelial cell OPG production

**Figure 4:**
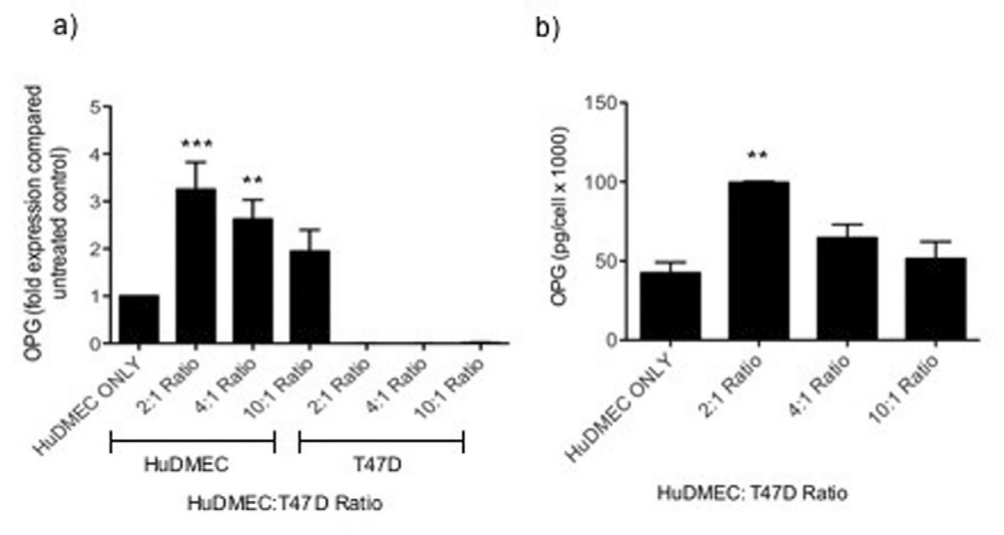
**Effect of tumour cell contact on endothelial cell OPG production**. HuDMECs were co-cultured with the T47D cell line at HuDMEC: T47D ratios of 2:1, 4:1 and 10:1 for 72 hours as described in materials and methods. HuDMECs were separated from the T47D cell line and gene expression measured in the separate cell populations using real-time quantitative PCR (a). OPG secretion was assessed using ELISA (b). For real-time quantitative PCR, values were normalised to GAPDH and are given as fold expression compared to untreated HuDMECs. Data are represented as mean ± S.E.M. from three independent experiments. ***, *p *< 0.001 compared to control cells (HuDMEC only); **, *p *< 0.01 compared to control.

An alternative mechanism by which OPG production by HuDMECs might be enhanced is through direct contact with tumour cells. Since clinical studies have demonstrated increased OPG expression in breast tumour endothelial cells, HuDMECs were co-cultured with the T47D breast cancer cell line, which did not produce detectable levels of OPG (data not shown). These co-cultures were established at HuDMEC: T47D ratios of 2:1, 4:1 and 10:1. Following a 72-hour incubation period, HuDMECs were separated from the T47D cells using CD31 Dynabeads and OPG gene expression assessed in each cell type. As demonstrated in figure 4(a), tumour cell contact significantly increased OPG gene expression levels compared to HuDMECs cultured alone. This was particularly noticeable at a 2:1 HuDMEC: T47D ratio, where OPG levels were increased 3-fold (*p *< 0.001). This was specific to HuDMECs as OPG gene expression was not detectable in the T47D cells following co-culture. The elevated levels of OPG expression were accompanied by increased HuDMEC OPG secretion. This increase was significant in co-cultures with a 2:1 HuDMEC: T47D ratio (100 pg/1000 cells) compared to HuDMECs cultured alone (42 pg/1000 cells) (*p *< 0.01) (Figure 4(b)). The increase in OPG expression seen in the co-cultures was dependent on direct cell-cell contact, as addition of conditioned medium from T47D monolayers had no effect on HuDMEC OPG levels (data not shown).

### Tumour cell contact-mediated HuDMEC OPG production involves NFkB and integrin α_ν_β_3_

**Figure 5:**
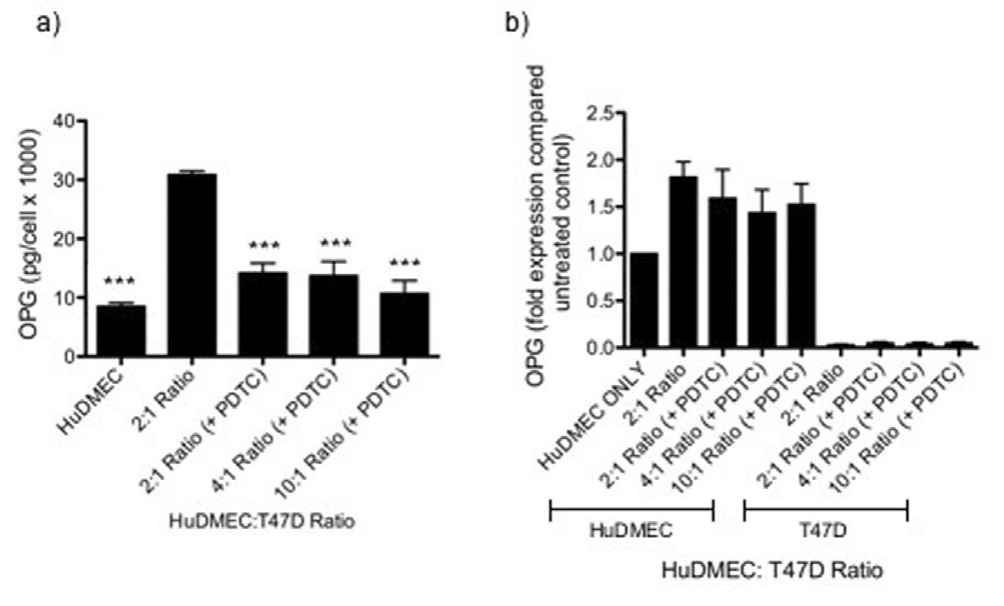
**Effect of NFκB inhibition on tumour cell contact mediated endothelial cell OPG production**. HuDMECs were co-cultured with the T47D cell line at HuDMEC: T47D ratios of 2:1, 4:1 and 10:1 for 72 hours in the presence or absence of the NFκB inhibitor PDTC as described in materials and methods. HuDMECs were separated from the T47D cell line and gene expression measured in the separate populations. OPG secretion was assessed using ELISA (a) and gene expression measured using real-time quantitative PCR (b). For real-time quantitative PCR, values were normalised to GAPDH and are given as fold expression compared to untreated HuDMECs. Data are represented as mean ± S.E.M. from three independent experiments. ***, *p *< 0.001 compared to HuDMEC: T47D 2:1 ratio co-culture without PDTC.

**Figure 6:**
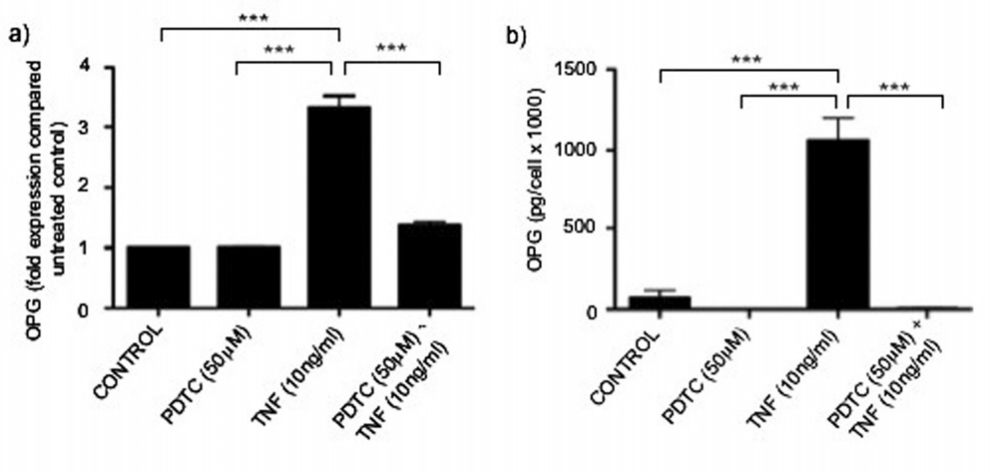
**Effect of NFκB inhibition on TNFα mediated endothelial cell OPG production**. HuDMECs were treated with the NFκB inhibitor PDTC (50 μM), TNFα (10 ng/ml) or TNFα (10 ng/ml) in conjunction with PDTC (50 μM) for 24 hours. RNA was extracted and gene expression quantified using real-time PCR (a). For real-time quantitative PCR, values were normalised to GAPDH and are given as fold expression compared to untreated HuDMECs. OPG secretion was measured in the conditioned medium using ELISA as described in materials and methods (b). Data are represented as mean ± S.E.M. from three independent experiments performed in triplicate. ***, *p *< 0.001.

Previous studies have demonstrated both the bacterial pathogen *Porphyromonas gingivalis *and the secreted glycoprotein osteopontin to increase endothelial cell OPG production in an NFκB-dependent manner [4,21]. To determine the involvement of NFkB in tumour cell contact-mediated HuDMEC production, co-cultures were established as previously and treated with the NFkB inhibitor PDTC (50 μM). As shown in figure 5(a), the tumour cell contact-mediated increase in HuDMEC OPG secretion was attenuated following NFκB inhibition with PDTC. Whilst untreated co-cultures at a 2:1 HuDMEC: T47D ratio secreted 30 pg/1000 cells of OPG, this was decreased by 53% to 14 pg/1000 cells in those treated with the PDTC (*p *< 0.001). In contrast, NFkB inhibition did not affect the tumour cell contact-mediated increase in HuDMEC OPG gene expression, suggesting NFκB involvement at the post-transcriptional level (Figure 5(b)). As a control, the effect of NFκB inhibition on TNFα induced HuDMEC OPG production was assessed. As demonstrated in figure 6, in contrast to the co-cultures, the TNFα mediated increase in both HuDMEC OPG gene expression and secretion was attenuated following treatment with PDTC. In terms of gene expression, PDTC significantly inhibited the TNFα mediated increase in OPG levels (*p *< 0.001). Similarly, whilst treatment of HuDMECs with TNFα significantly increased OPG secretion from 74.53 pg/1000 cells to 1065 pg/1000 cells (*p *< 0.001), in the presence of PDTC this was significantly decreased to 5.76 pg/1000 cells (*p *< 0.001).

**Figure 7:**
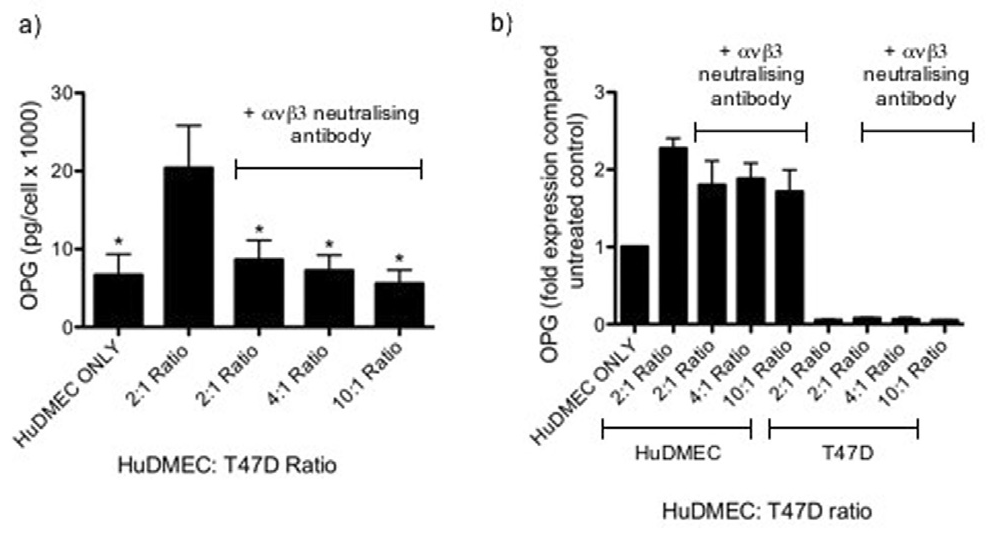
**Effect of integrin α_ν_β_3 _neutralisation on tumour cell contact mediated endothelial cell OPG production**. HuDMECs were co-cultured with the T47D cell line at HuDMEC: T47D ratios of 2:1, 4:1 and 10:1 for 72 hours, in the presence or absence of an integerin α_ν_β_3 _neutralising antibody as described in materials and methods. HuDMECs were separated from the T47D cell line and gene expression measured in the separate cell populations. OPG secretion was assessed using ELISA (a) and gene expression measured using real-time quantitative PCR (b). For real-time quantitative PCR, values were normalised to GAPDH and are given as fold expression compared to untreated HuDMECs. Data represented as mean ± S.E.M. from three independent experiments. *, *p *< 0.05 compared to HuDMEC: T47D 2:1 ratio co-culture without the integrin α_ν_β_3 _neutralising antibody.

In endothelial cells, NFkB-dependent OPG production has previously been found to involve integrin α_ν_β_3 _ligation [4]. We therefore investigated whether this mechanism could be involved in tumour cell contact-mediated HuDMEC OPG production. To address this issue, co-cultures were established as previously and treated with an integrin α_ν_β_3 _neutralising antibody (10 μg/ml) for 72 hours. As demonstrated in figure 7(a), the tumour cell contact-mediated increase in OPG secretion by HuDMEC was inhibited following integrin α_ν_β_3 _neutralisation. Whilst untreated co-cultures at a 2:1 HuDMEC:T47D ratio secreted 20 pg/1000 cells of OPG, this was decreased by over 50% to 9 pg/1000 cells in those treated with the integrin α_ν_β_3 _neutralising antibody (*p *< 0.05). As was observed for NFκB inhibition, integrin α_ν_β_3 _neutralisation did not affect the tumour cell contact-mediated increase in HuDMEC OPG gene expression, suggesting involvement at the post-transcriptional level (Figure 7(b)).

## Discussion

This study has looked at two key aspects of OPG in endothelial cell biology, with a particular focus on the potential role of OPG in tumour angiogenesis. Firstly, the involvement of the VEGFR2 signalling pathway in OPG-mediated endothelial cell tube formation has been eliminated, narrowing the search for the mechanism involved in this process. Secondly, we have demonstrated the requirement for tumour cell contact in increasing OPG production in endothelial cells, a process partly dependent on integrin α_ν_β_3 _ligation and activation of the NFκB pathway. These findings could give an important insight into the processes involved in the proposed increased OPG levels within the tumour microenvironment.

OPG has previously been found to induce endothelial cell tube formation in an *in vitro *matrigel model of angiogenesis [3]. However, the mechanisms responsible for this are unknown. In this study, OPG in combination with VEGF was found to have an additive effect on endothelial cell tube formation compared to either of the two treatments alone. This additive effect does not appear to be exclusive to OPG, since other studies have demonstrated similar observations in endothelial cells treated with a combination of VEGF and bFGF [22,23]. Therefore, these results suggest that within the tumour microenvironment, where a variety of pro-angiogenic factors are likely to be present, these can act in concert to promote tumour angiogenesis. Additionally, our results show that OPG is unlikely to induce endothelial cell tube formation via the same mechanism as VEGF. Investigating this further, we found that neutralisation of the VEGF receptor VEGFR2 had no effect on OPG-mediated endothelial cell tube formation, whilst significantly inhibiting that of VEGF. Although VEGF is able to exert its effects through a number of receptors, our results combined suggest it is probable that OPG acts via an alternative mechanism.

One possible mechanism could be via the interaction of OPG with heparan sulphate proteoglycans of the syndecan family, which have previously been reported to be involved in OPG-mediated monocyte chemotaxis [24]. Additionally, the observation that the syndecan binding peptide AG73 can induce endothelial cell tube formation suggests the involvement of syndecans in this process [25]. In our study recombinant Fc-conjugated OPG was used, the heparin binding ability of which is suggested to be compromised [26]. However, other studies have shown both OPG and OPG-Fc to bind to cells in an apparently heparan sulphate-dependent manner [27,28]. Therefore the potential involvement of the syndecans in OPG-mediated endothelial cell tube formation cannot be completely dismissed. OPG has previously been observed to bind to the anti-angiogenic protein thrombospondin-1 (TSP-1) [29]. TSP-1 is released by endothelial cells and inhibition of TSP-1 activity using blocking antibodies promotes endothelial cell tube formation [30]. Therefore, another consideration could be that OPG binds to and inhibits TSP-1 activity, resulting in an increase in endothelial cell tube formation. Additionally, it would be of interest to assess the role of the integrins in this process, given recent evidence of their role in OPG-mediated endothelial cell migration [14].

We have also demonstrated that tumour cell contact, but not the pro-angiogenic factors VEGF, bFGF or TGFβ, enhance OPG production in microvascular endothelial cells. In agreement with previously published studies, we found that the proinflammatory cytokine TNFα increased HuDMEC OPG levels, supporting that the cells used in this study retained the responsiveness to cytokines [18-20]. The role of TGFβ in regulating OPG levels has been studied in other cell types, but the effects appear to depend on the cell type used. Whilst TGFβ can increase OPG production in osteoblasts and bone marrow stromal cells, levels have been found to be decreased in human vascular smooth muscle cells [31-33]. This suggests along with our findings, that with regards to vascular cells, the role of TGFβ in increasing OPG levels is negligible, at least *in vitro*.

Although VEGF has previously been found to increase OPG production in endothelial cells in combination with TNFα [34], no study until now has assessed the effects of VEGF alone. Our findings demonstrate that VEGF administered alone has no effect on endothelial cell OPG production. Tumours often exhibit an inflammatory component and it may be possible under those conditions that VEGF could act in conjunction with TNFα to augment OPG levels. However, in studies demonstrating increased endothelial cell OPG expression in breast cancer tissues, no notable inflammation was observed, suggesting alternative mechanisms exist [3].

Another way in which endothelial cell OPG expression may be enhanced within the tumour microenvironment is through direct tumour cell contact. This is the first study to demonstrate that contact between breast cancer cells and endothelial cells results in increased gene and protein levels of endothelial OPG, a process that appears to depend on integrin α_ν_β_3 _ligation and activation of the transcription factor NFκB. Interestingly, inhibition of NFκB and integrin α_ν_β_3 _did not induce tumour cell contact mediated increases in OPG gene expression in HuDMEC, indicating their involvement at the post transcriptional level, possibly by affecting mechanisms involved in mediating OPG secretion. Observations that tumour cell conditioned medium had no effect on endothelial cell OPG levels suggests this process requires direct tumour cell contact. However, it cannot be ruled out that contact between tumour and endothelial cells results in the secretion of as yet unidentified factors from tumour cells that are capable of inducing endothelial cell OPG production. One potential candidate is the secreted glycoprotein and integrin α_ν_β_3 _ligand osteopontin, which has previously been observed to increase endothelial OPG levels in an NFκB dependent manner following integrin α_ν_β_3 _binding [4]. Interestingly, osteopontin expression has been found to be increased in prostate cancer cells following their direct cell contact with bone marrow stromal cells [35]. Therefore it is feasible to consider that osteopontin could be increased in other tumour-derived cell lines following direct cell contact.

## Conclusion

To conclude, this study has demonstrated that the VEGF signalling pathway is not involved in mediating the pro-angiogenic effects of OPG on endothelial cells *in vitro*, thus narrowing the search for the mechanism by which these processes occur. Secondly, this is the first study to show that endothelial cell OPG is increased following direct contact with breast tumour cells via a mechanism that depends partly on integrin α_ν_β_3 _ligation and NFκB activation. Therefore, observed increases of endothelial OPG in malignant tissue, particularly that of breast cancer, could be a result of direct interactions between tumour and endothelial cells within the tumour microenvironment. Our data support a potential role for endothelial cell-derived OPG in tumour angiogenesis.

## Methods

### Cell culture

Human dermal microvascular endothelial cells (HuDMECs) were extracted from excess tissue, with the patients' informed consent, following routine breast surgery (kindly provided by Professor M. W. Reed, Royal Hallamshire Hospital, Sheffield, UK; Ethics number: SSREC98/198). Cells were grown in T75 tissue culture flasks in basic medium (EBM2) containing growth supplements (EGM-2MV, Lonza, Wokingham, UK). All cells used in this study were between passages 3 and 7. The T47D human breast cancer cell line (American Type Culture Collection, Manassas, Virginia, USA) was maintained in RPMI 1640 (Invitrogen, Paisley, UK) supplemented with foetal calf serum (10%) and L-glutamine (2 mM).

### Tubule formation assay

Recombinant human VEGF 165 and recombinant human OPG/Fc chimera were purchased from R & D Systems (Abingdon, UK). HuDMECs were trypsinised, washed with PBS, counted and resuspended in EBM2 containing 1% FBS with VEGF (1 ng/ml), OPG (10 ng/ml) or VEGF (1 ng/ml) in combination with OPG (10 ng/ml). For experiments investigating the effect of VEGFR2 neutralisation HuDMECs were resuspended in EBM2 containing VEGF (10 ng/ml), OPG (100 ng/ml), VEGF (10 ng/ml) in combination with 1 μg/ml VEGFR2 neutralising antibody (R & D systems (Abingdon, UK) or OPG (100 ng/ml) in combination with 1 μg/ml VEGFR2 neutralising antibody. Cells were seeded at a density of 2 × 10^4 ^cells/well onto solidified growth factor reduced Matrigel (BD Biosciences, Oxford, UK) in 24 well plates and incubated at 37°C for 8 hours. Tubular/cord-like networks were visualised with an inverted light microscope (4× objective), photographed and tube formation assessed by counting branch point number, using 3 fields per well.

### Treatment of HuDMECs with pro-angiogenic factors

Recombinant human TNFα, recombinant human VEGF 165, bFGF and recombinant human TGFβ were all purchased from R & D systems (Abingdon, UK). HuDMECs were seeded onto 24 well plates at a density of 1 × 10^4 ^cells/well in EGM-2MV containing TNFα (1, 10 and 50 ng/ml), VEGF (1,10 and 25 ng/ml), bFGF (10–25 ng/ml) or TGFβ (5 and 10 ng/ml). After 24 hours incubation, conditioned medium was removed for ELISA and RNA extracted from treated cells for real-time quantitative PCR.

### Co-culture of HuDMECs and T47D cells

HuDMECs and T47D cells were seeded into T25 tissue culture flasks at HuDMEC: T47D ratios of 2:1, 4:1 and 10:1 and cultured in basic medium (EBM2) containing growth supplements (EGM-2MV). After 72 hours incubation, conditioned medium was removed for ELISA and the co-cultures trypsinised and centrifuged at 1,000 rpm for 5 minutes. The pellet was resuspended in EGM-2MV containing washed CD31 coated Dynabeads (25 μl per ml of cell suspension) (Invitrogen, Paisley, UK) and incubated at 4°C with end over end rotation. After 20 minutes, the solution was placed in a Dynal MPC™ magnet for 3 minutes, allowing the endothelial cells and beads to form a pellet. The supernatant containing T47D cells was transferred to a separate tube. This step was repeated a further 2 times, after which the RNA was extracted from both cell types for real-time quantitative PCR.

For studies assessing the effect of NFκB inhibition, cells were cultured as stated above in the presence or absence of the NFκB inhibitor ammonium pyrrolidinedithiocarbamate (PDTC) (50 μM) (Sigma-Aldrich, Poole, UK). Similarly, when studying the effects of integrin α_ν_β_3 _inhibition, cells were cultured as above in the presence or absence of an integrin α_ν_β_3 _neutralising antibody (10 μg/ml) (Millipore, Co Durham, UK).

### Determination of OPG concentration by ELISA

The concentration of OPG in the culture medium was determined using an ELISA method. Briefly, 96 well plates were coated with 2 μg/ml mouse monoclonal anti-human OPG (R&D Systems, Abingdon, UK). An OPG standard curve was created using recombinant human OPG (R&D Systems) at concentrations ranging from 31.2 to 1000 pg/ml. The secondary antibody, biotinylated anti-human OPG (R&D Systems), was used at a concentration of 200 ng/ml. OPG protein levels were detected using streptavidin-horseradish peroxidase, followed by addition of substrate solution (R & D Systems). After a 30 minute incubation period, the reaction was stopped using 2 M H_2_SO_4 _and the plate read at 450 nm on a Dynatech plate reader using revelation software.

### Real-time quantitative RT-PCR

RNA was extracted from cultured cells using TRI REAGENT™ (Sigma, Poole, UK) and reverse-transcribed using Superscript II Reverse Transcriptase (Invitrogen, Paisley, UK). Relative expression of OPG was compared to GAPDH using probes and primers supplied in the GAPDH and OPG TaqMan^® ^Gene Expression Assays (Applied Biosystems, Warrington, UK). Real-time PCR amplification of cDNA was performed using TaqMan^® ^Universal PCR mastermix (Applied Biosystems) on an ABI7900 PCR system and results analysed using SDS 2.0 software (Applied Biosystems).

### Statistical Analysis

Statistical analysis was performed using GraphPad PRISM^® ^(version 5.0a). One-way analysis of variance (ANOVA) and the Newman-Keuls test for post-hoc comparisons were used to test for significant differences between groups.

## Competing interests

The authors declare that they have no competing interests.

## Authors' contributions

IH supervised the study, participated in its design and coordination, and helped draft/review the manuscript. NJ participated in the design of the study. PR performed experimental work, participated in the design of the study and wrote the manuscript.
